# Root Filling

**Published:** 1900-02

**Authors:** W. R. Rathbone

**Affiliations:** Cuers, Texas.


ARTICLE V.

### ROOT FILLING.

DR. W. R. RATHBONE, CUERS, TEXAS.

Dr. Maynard was the first to show, over fifty years ago, that most of the trouble resulting from the presence of a tooth in the mouth after the destruction of the nerve arose from the irritation produced by the matter contained in the pulp canal. To prevent this occurrence, therefore, he proposes filling the canals in such a manner as to completely exclude everything else.

Therefore, if the pulp has been exposed and is giving trouble, in my judgment it is the wisest, safest and surest plan to devitalize and remove same, this being the only course to pursue with a fair promise for satisfactory results.

The virtue of this operation was for a long time questioned by many, but same has now become very common, and is practiced by all skillful dentists.

Some operators drill out these canals, and thus give them the same diameter from the orifice at the pulp chamber to the apex of the root. This I consider a very dangerous operation, as the instrument may pass through the side of the root—especially should the root be curved. In a great majority of cases thoroughly cleansing the canal of all debris and decomposed dentine and slightly reaming out the orifice of the canal will be sufficient; which ever method is pursued care is necessary that the instrument is not carried through the foramen.

After having reached this stage we are ready for the selection of a proper filling material, etc.

A great many arguments can be advanced both pro and con in reference to most all root fillings, but from an experience of more than thirteen years I find I have been very successful in fillng roots with oxychloride of zinc and cotton. Prof. J. Foster Flagg is a great champion of cotton as a partial root filling, and has used it very extensively. Absorbent cotton is not as desirable as the crude uncarded cotton wool. Dr. Flagg cites as a proof of the "impermeability of this material, when properly packed, that bales of cotton which have floated in sea water for long periods, when opened, show no evidence of moisture in their interior."

When you have, through patience and perseverance, accomplished all that is possible in the effort at the removal of nerve filaments, syringe well with water, dry thoroughly, and, as far as you can, saturate with campho-phenique. It being a well-known fact that oxychloride of zinc preparations are irritating to the vital tissues, it is therefore, necessary that a barrier be placed between the paste and apex of the root. For this purpose I usually take a small piece of cotton not larger than a pin head, and moisten a little, and coat with campho phenique powder, then thoroughly pack this as near the apical foramen as possible. Ii is said that when the freshly mixed chloride comes in contact with the cotton it becomes converted into amyloid, which hermetically and permanently seals the apical foramen. The same change of course takes place in the cotton upon which the oxychloride is carried into position. A thin paste of oxychloride is mixed, into which you place a rope of loosely rolled cotton fibre until well saturated with same. The small end then carried into the canal up to the guard at apex with small round, smooth points very suitably made from piano wire and fitted into broache handles. When filled the surplus cement can be pressed out with bibulous paper. A tooth thus filled will not show the fibres of the cotton upon excavations into the cement after it has become hardened. I do not claim the cotton adds any to the properties of the oxychloride of zinc, but on the other hand I do not think it detrimental, and that the cotton fibre acts as a vehicle for the oxychloride, thereby avoiding the danger of leaving a vacuum in the canals caused by air bubbles.—*Texas Dental Journal.*

